# Determinants of pre-vaccination antibody responses to SARS-CoV-2: a population-based longitudinal study (COVIDENCE UK)

**DOI:** 10.1186/s12916-022-02286-4

**Published:** 2022-02-22

**Authors:** Mohammad Talaei, Sian Faustini, Hayley Holt, David A. Jolliffe, Giulia Vivaldi, Matthew Greenig, Natalia Perdek, Sheena Maltby, Carola M. Bigogno, Jane Symons, Gwyneth A. Davies, Ronan A. Lyons, Christopher J. Griffiths, Frank Kee, Aziz Sheikh, Alex G. Richter, Seif O. Shaheen, Adrian R. Martineau

**Affiliations:** 1 Wolfson Institute of Population Health, Barts and The London School of Medicine and Dentistry, Queen Mary University of London, London, UK; 2 Institute of Immunology and Immunotherapy, College of Medical and Dental Sciences, University of Birmingham, Birmingham, UK; 3 Blizard Institute, Barts and The London School of Medicine and Dentistry, Queen Mary University of London, London, UK; 4 Asthma UK Centre for Applied Research, Queen Mary University of London, London, UK; 5 Jane Symons Media, London, UK; 6 Population Data Science, Swansea University Medical School, Singleton Park, Swansea, UK; 7 Centre for Public Health Research (NI), Queen’s University Belfast, Belfast, UK; 8 Usher Institute, University of Edinburgh, Edinburgh, UK

**Keywords:** SARS-CoV-2, Serology, Ethnicity, Diet, Micronutrients, Lifestyle, Exercise, Obesity, Alcohol, Occupation

## Abstract

**Background:**

Prospective population-based studies investigating multiple determinants of pre-vaccination antibody responses to SARS-CoV-2 are lacking.

**Methods:**

We did a prospective population-based study in SARS-CoV-2 vaccine-naive UK adults recruited between May 1 and November 2, 2020, without a positive swab test result for SARS-CoV-2 prior to enrolment. Information on 88 potential sociodemographic, behavioural, nutritional, clinical and pharmacological risk factors was obtained through online questionnaires, and combined IgG/IgA/IgM responses to SARS-CoV-2 spike glycoprotein were determined in dried blood spots obtained between November 6, 2020, and April 18, 2021. We used logistic and linear regression to estimate adjusted odds ratios (aORs) and adjusted geometric mean ratios (aGMRs) for potential determinants of SARS-CoV-2 seropositivity (all participants) and antibody titres (seropositive participants only), respectively.

**Results:**

Of 11,130 participants, 1696 (15.2%) were seropositive. Factors independently associated with  higher risk of SARS-CoV-2 seropositivity included frontline health/care occupation (aOR 1.86, 95% CI 1.48–2.33), international travel (1.20, 1.07–1.35), number of visits to shops and other indoor public places (≥ 5 vs. 0/week: 1.29, 1.06–1.57, P-trend = 0.01), body mass index (BMI) ≥ 25 vs. < 25 kg/m^2^ (1.24, 1.11–1.39), South Asian vs. White ethnicity (1.65, 1.10–2.49) and alcohol consumption ≥15 vs. 0 units/week (1.23, 1.04–1.46). Light physical exercise associated with  lower risk (0.80, 0.70–0.93, for ≥ 10 vs. 0–4 h/week). Among seropositive participants, higher titres of anti-Spike antibodies associated with factors including BMI ≥ 30 vs. < 25 kg/m^2^ (aGMR 1.10, 1.02–1.19), South Asian vs. White ethnicity (1.22, 1.04–1.44), frontline health/care occupation (1.24, 95% CI 1.11–1.39), international travel (1.11, 1.05–1.16) and number of visits to shops and other indoor public places (≥ 5 vs. 0/week: 1.12, 1.02–1.23, P-trend = 0.01); these associations were not substantially attenuated by adjustment for COVID-19 disease severity.

**Conclusions:**

Higher alcohol consumption and lower light physical exercise represent new modifiable risk factors for SARS-CoV-2 infection. Recognised associations between South Asian ethnic origin and obesity and higher risk of SARS-CoV-2 seropositivity were independent of other sociodemographic, behavioural, nutritional, clinical, and pharmacological factors investigated. Among seropositive participants, higher titres of anti-Spike antibodies in people of South Asian ancestry and in obese people were not explained by greater COVID-19 disease severity in these groups.

**Supplementary Information:**

The online version contains supplementary material available at 10.1186/s12916-022-02286-4.

## Background

The COVID-19 pandemic has caused more than 220 million recorded infections and over 4.5 million recorded deaths [1], with these figures representing only a portion of the true burden [2]. Large population-based studies have identified various risk factors for SARS-CoV-2 infection, including non-White ethnicity and lower educational attainment [3–5]. However, the vast majority of studies have been based on routine real-time reverse transcription PCR (RT-PCR) testing in healthcare settings or in the community; consequently, they are potentially open to collider bias, as the probability of being tested for infection can itself depend on the risk factors under investigation [6]. Access to testing has also changed across the course of the pandemic [7], meaning earlier studies were more likely to focus on people with symptomatic disease or a history of travel, or on specific populations such as healthcare workers.

Serological population-based studies offer a different approach by testing members of a population uniformly, including people who might not be captured by routine testing. This approach not only reduces the risk of collider bias but also can uncover previously undetected asymptomatic infections. Inclusion of asymptomatic SARS-CoV-2 infections in the analysis of risk factors is crucial, as asymptomatic individuals have been found to be as infectious as those with symptoms [8]. Serology studies also offer the opportunity to identify determinants of anti-SARS-CoV-2 antibody titres, which are a recognised correlate of protection against future infection [9, 10].

The largest population-based serology studies done to date have explored several sociodemographic and clinical risk factors, but have not considered risk factors related to lifestyle, diet, or levels of physical activity [3–5, 11, 12]. These studies have focused on IgG antibodies alone [11, 12] or relied on immunoassays with low sensitivity [12], potentially missing infections. They have also tended to be cross-sectional in design, so that reverse causality could potentially explain associations between symptomatic seropositivity and modifiable risk factors. Additionally, studies investigating determinants of antibody titres have focused on specific populations such as healthcare workers [13, 14], limiting the generalisability of their findings.

We therefore undertook a prospective population-based study to uncover determinants of SARS-CoV-2 seropositivity and antibody titres, combining high statistical power with detailed assessment of sociodemographic, clinical and behavioural risk factors, and supported by an assay with proven sensitivity for detection of SARS-CoV-2 antibodies in non-hospitalised adults with mild or moderate COVID-19 [15].

## Methods

### Study design and participants

COVIDENCE UK is a prospective, longitudinal, population-based observational study of COVID-19 in the UK population (www.qmul.ac.uk/covidence) [16]. Inclusion criteria were age 16 years or older and UK residence at enrolment, with no exclusion criteria. Participants were invited via a national media campaign to complete an online baseline questionnaire to capture information on potential symptoms of COVID-19 experienced since February 1, 2020, results of any COVID-19 tests, and details of a wide range of potential risk factors for COVID-19 (Additional file 1: Table S1). Online monthly follow-up questionnaires captured incident test-confirmed COVID-19 and symptoms of acute respiratory infection (Additional file 1: Table S2). The study was launched on May 1, 2020.

The antibody study described here was introduced as an approved protocol amendment (amendment 3; November, 2020). Participants enrolled before the amendment were invited via email to participate in the antibody study and to give additional consent. As part of the antibody study, participants were invited to participate in serology testing from November, 2020. For this analysis, we included all participants who enrolled in the study between May 1 and November 2, 2020, partaking in serology testing who were not vaccinated against COVID-19 or who provided their dried blood spot sample on or before the date of their first COVID-19 vaccination. This paper reports findings from analysis of data collected up to April 18, 2021.

COVIDENCE UK was sponsored by Queen Mary University of London and approved by Leicester South Research Ethics Committee (ref 20/EM/0117). It is registered with ClinicalTrials.gov (NCT04330599).

### Procedures

Antibody study participants were sent a kit containing instructions, lancets, and blood spot collection cards, to be posted back to the study team. Once returned, the samples were logged by the study team and sent in batches to the Clinical Immunology Service at the Institute of Immunology and Immunotherapy of the University of Birmingham (Birmingham, UK). Up to two more test kits were offered to participants whose initial samples were found to be insufficient for testing. Blood spot samples were taken from November 6, 2020, to April 18, 2021.

Semi-quantitative determination of antibody titres in dried blood spot eluates was done using a commercially available ELISA that measures combined IgG, IgA, and IgM (IgGAM) responses to the SARS-CoV-2 trimeric spike glycoprotein (product code MK654; The Binding Site [TBS], Birmingham, UK). The SARS-CoV-2 spike used is a soluble, stabilised, trimeric glycoprotein truncated at the transmembrane region [17, 18]. This assay has been CE-marked with 98.3% (95% CI 96.4–99.4) specificity and 98.6% (92.6–100.0) sensitivity following RT-PCR-confirmed mild-to-moderate COVID-19 that did not result in hospitalisation [15]. A cut-off ratio relative to the TBS cut-off calibrators was determined by plotting 624 pre-2019 negatives in a frequency histogram. A cut-off coefficient was then established for IgGAM (1.31), with ratio values classed as positive (≥ 1) or negative (< 1). Dried blood spots were pre-diluted at a 1:40 dilution with 0.05% PBS-Tween using a Dynex Revelation automated absorbance microplate reader (Dynex Technologies; Chantilly, VA, USA). Plates were developed after 10 min using 3,3′,5,5′-tetramethylbenzidine core and orthophosphoric acid used as a stop solution (both TBS). Optical densities at 450 nm were measured using the Dynex Revelation. Results of ELISA for detection of anti-Spike antibodies in dried blood spot eluates have previously been shown to have almost perfect agreement with those performed on serum (Cohen’s kappa = 0.83) [15].

### Outcomes

Study outcomes were presence versus absence of antibodies against SARS-CoV-2 (binary outcome assessed in all participants who did not report having tested positive for SARS-CoV-2 infection via RT-PCR or lateral flow test before enrolment) and antibody titres (continuous outcome measured in all seropositive participants).

### Independent variables

Eighty-eight putative risk factors for SARS-CoV-2 infection were selected a priori, covering sociodemographic, occupational and lifestyle factors; longstanding medical conditions and prescribed medication use; Bacille Calmette Guérin and measles, mumps, and rubella vaccine status; and diet and supplemental micronutrient intake (Additional file 1: Tables S1, S2). These factors, which were obtained from the baseline questionnaire, were included as independent variables in our models. To produce patient-level covariates for each class of medications investigated, participant responses were mapped to drug classes listed in the British National Formulary or the DrugBank and Electronic Medicines Compendium databases if not explicitly listed in the British National Formulary, as previously described [16]. Index of Multiple Deprivation (IMD) 2019 scores were assigned according to participants’ postcodes, and categorised into quartiles. Duration of follow-up was defined as the number of days between the date of enrolment and the date of dried blood spot collection.

### Statistical analysis

Using the Stata powerlog program, we estimated that a minimum sample size of 10,964 would be required to detect a difference of at least 2% in the proportion of exposed vs. unexposed participants experiencing a given binary outcome [equivalent to an odds ratio (OR) of 1.08], with 90% power, for a binary exposure with maximum variability (probability 0.50 changing to 0.52) and a moderate correlation (*R*^2^ = 0.4) with other variables in a logistic regression model, using a two-sided test and 5% significance. The antibody study was a pragmatic study including all participants meeting the inclusion criteria, with no sample size specified.

Logistic regression models were used to estimate ORs and 95% CIs for potential determinants of SARS-CoV-2 seropositivity. Linear regression models with robust standard errors were used to estimate geometric mean ratios (GMRs) and 95% CIs for potential determinants of log-transformed antibody titres in seropositive participants. We first estimated ORs and GMRs in minimally adjusted models, and carried forward factors independently associated with each outcome at the 10% significance level to fully adjusted models. Both the minimally adjusted and fully adjusted models were controlled for age (< 30 years, 30 to < 40 years, 40 to < 50 years, 50 to < 60 years, 60 to < 70 years, and ≥ 70 years), sex (male vs. female) and duration of follow-up (days). We calculated *p* for trend for ordinal variables by re-running the regressions treating each ordinal variable in turn as continuous. Analyses were done for all participants with available data; missing data were not imputed. Correction for multiple comparisons was not applied, on the grounds that we were testing a priori hypotheses for all risk factors investigated [19].

In a sensitivity analysis, we excluded participants from the seropositivity analysis who were classified as having had probable COVID-19 before enrolment on the basis of self-reported symptoms, using the symptom algorithm described and validated by Menni and colleagues [20].

As antibody titres have been found to be associated with disease severity [13, 21], we did an exploratory analysis to investigate the extent to which COVID-19 severity might explain associations between independent variables and antibody titres, by including this as an explanatory variable in the titre analysis. COVID-19 severity was classified into three groups: ‘asymptomatic’ (non-hospitalised seropositive participants, who either did not report any symptoms of acute respiratory infection or whose symptoms were classified as having < 50% probability of being due to COVID-19, using the symptom algorithm by Menni and colleagues [20]), ‘symptomatic non-hospitalised’ (non-hospitalised seropositive participants who reported symptoms of acute respiratory infection that were classified as having ≥ 50% probability of being due to COVID-19, using the symptom algorithm [20]) and ‘hospitalised’ (seropositive participants who were hospitalised for treatment of COVID-19).

We present descriptive statistics as *n* (%), mean (SD), or median (IQR). Statistical analyses were done using Stata (version 14.2; StataCorp, College Station, TX, USA).

### Role of the funding source

The study funders had no role in the study design, data analysis, data interpretation, or writing of the report.

## Results

**Fig. 1:**
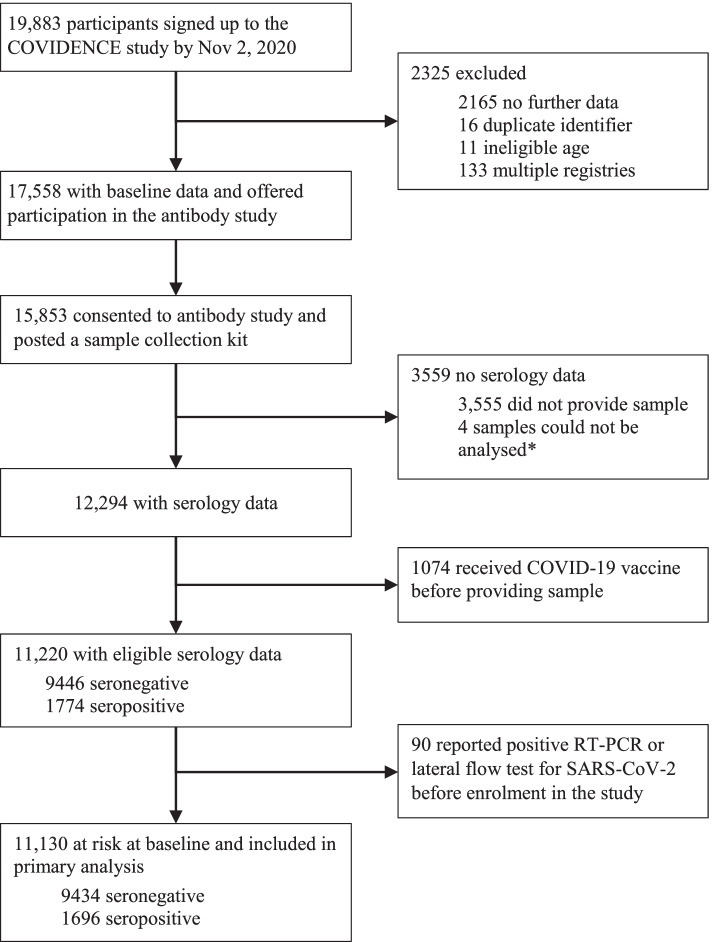
Study profile. Asterisk indicates the following: 103 participants provided insufficient samples, but 99 were successfully analysed upon repeat test

Serology data were available for 12,294 of the 15,853 participants who consented to participate in the antibody study. We excluded data from 1074 participants who had been vaccinated against SARS-CoV-2 before providing their dried blood spot sample (Fig. 1). Of the 11,220 participants included, 1774 (15.8%) tested positive for SARS-CoV-2 antibodies. For the analysis of determinants of seropositivity, we excluded 90 (0.8%) participants who reported a positive RT-PCR or lateral flow test result for SARS-CoV-2 infection before enrolment, leaving a sample size of 11,130 participants with 1696 seropositive cases (Fig. 1). Selected baseline characteristics of included participants are shown in Table 1. 70.1% of participants were female, and 95.7% identified their ethnicity as White, with median age of 62.3 years (IQR 52.9–68.7; Table 1).

**Table 1:** Baseline characteristics of participants included in seropositivity analysis

	Characteristics (***N*** = 11,130)
**Sociodemographic, occupational and lifestyle factors**	
Age (years)	62.3 (52.9–68.7)
< 30	323 (2.9%)
30 to < 40	651 (5.8%)
40 to < 50	1246 (11.2%)
50 to < 60	2545 (22.9%)
60 to < 70	4098 (36.8%)
≥ 70	2267 (20.4%)
Sex	
Female	7806 (70.1%)
Male	3324 (29.9%)
Ethnicity	
Black, African, Caribbean or Black British	49 (0.4%)
South Asian^a^	145 (1.3%)
Mixed, multiple or other ethnic groups	285 (2.6%)
White	10,651 (95.7%)
Country of residence	
England	9835/11,129 (88.4%)
Northern Ireland	188/11,129 (1.7%)
Scotland	694/11,129 (6.2%)
Wales	412/11,129 (3.7%)
Housing	
Owns own home	7059/11,129 (63.4%)
Mortgage	2716/11,129 (24.4%)
Privately renting	654/11,129 (5.9%)
Renting from council	318/11,129 (2.9%)
Other	382/11,129 (3.4%)
Number of people per bedroom	
≤ 0.5	4382/11,059 (39.6%)
> 0.5 to < 1	3085/11,059 (27.9%)
1 to < 2	3355/11,059 (30.3%)
≥ 2	237/11,059 (2.1%)
Shares home with	
Pre-school children (0–4 years)	362/11,106 (3.3%)
Schoolchildren (5–15 years)	1227/11,101 (11.1%)
Working-age adult (16–64 years)	5748/11,101 (51.8%)
Household income sufficient for basic needs	
Yes	10,409/11,129 (93.5%)
Mostly	390/11,129 (3.5%)
Sometimes	80/11,129 (0.7%)
No	250/11,129 (2.2%)
Claiming Universal Credit	275/11,098 (2.5%)
IMD decile	7 (5–9)
Occupational status	
Employed	3662 (32.9%)
Self-employed	1048 (9.4%)
Retired	5284 (47.5%)
Furloughed	282 (2.5%)
Unemployed	186 (1.7%)
Student	174 (1.6%)
Other	494 (4.4%)
Frontline worker	1700/11,112 (15.3%)
Health or social care	499/11,112 (4.5%)
Other	1201/11,112 (10.8%)
Highest educational level attained	
Primary or secondary	1194/11,123 (10.7%)
Higher or further education (A levels or BTEC)	1600/11,123 (14.4%)
College or university	4961/11,123 (44.6%)
Postgraduate degree	3368/11,123 (30.3%)
Tobacco smoking status	
Never-smoker	6281 (56.4%)
Ex-smoker	4325 (38.9%)
Current smoker	524 (4.7%)
Regular environmental tobacco smoke exposure	203/11,128 (1.8%)
Vaping status	
Never-vaper	10,463/11,099 (94.3%)
Ex-vaper	339/11,099 (3.1%)
Current vaper	297/11,099 (2.7%)
Alcohol (units per week)	
None	2986/11,129 (26.8%)
1–7	3932/11,129 (35.3%)
8–14	2249/11,129 (20.2%)
≥ 15	1962/11,129 (17.6%)
Does any vigorous physical exercise	7001/11,102 (63.1%)
1–3 h per week	4142/11,102 (37.3%)
≥ 4 h per week	2859/11,102 (25.8%)
Duration of follow-up (days)	178 (26–419)
**Medical conditions and prescribed medication use**	
Self-reported general health	
Excellent	2291/11,129 (20.6%)
Very good	4435/11,129 (39.9%)
Good	2939/11,129 (26.4%)
Fair	1147/11,129 (10.3%)
Poor	317/11,129 (2.8%)
BMI, kg/m^2^	26.2 (5.3)
< 25	5424/11,106 (48.8%)
25–30	3586/11,106 (32.3%)
> 30	2096/11,106 (18.9%)
Comorbidities	
Arterial disease^b^	552 (5.0%)
Asthma	1800 (16.2%)
Atopy^c^	2861 (25.7%)
Autoimmune disease^d^	965 (8.7%)
Cancer	1036 (9.3%)
Past (cured or in remission)	945 (8.5%)
Present (active treatment)	91 (0.8%)
COPD	213 (1.9%)
Diabetes or pre-diabetes	858 (7.7%)
Pre-diabetes	332/11,118 (3.0%)
Type 1	80/11,118 (0.7%)
Type 2	434/11,118 (3.9%)
Heart disease^e^	408 (3.7%)
Hypertension	2379 (21.4%)
Immunodeficiency^f^	64 (0.6%)
Kidney disease	214 (1.9%)
Major neurological conditions^g^	287 (2.6%)

Data are *n* (%), *n*/*N* (%), mean (SD), or median (IQR)

*BTEC* Business and Technology Education Council, *BMI* body mass index, *COPD* chronic obstructive pulmonary disease, *IMD* Index of Multiple Deprivation

^a^South Asian indicates people who self-identified their ethnic origin as Indian, Pakistani or Bangladeshi

^b^Ischaemic heart disease, peripheral vascular disease or cerebrovascular disease

^c^Hay fever/allergic rhinitis or atopic eczema/dermatitis

^d^Including rheumatoid arthritis, multiple sclerosis, lupus, Crohn’s disease, ulcerative colitis and psoriasis

^e^Coronary artery disease or heart failure

^f^HIV, primary immunodeficiency disorder or other immunodeficiencies

^g^Stroke, transient ischaemic attack, dementia, Parkinson’s disease, multiple sclerosis or motor neuron disease

After adjustment for age, sex and duration of follow-up, 25 factors were independently associated with risk of SARS-CoV-2 seropositivity with *p* < 0.10 (Table 2). Additional file 1: Table S3 shows factors with no evidence of association. When the former factors were included together in a fully adjusted model, we observed that South Asian ethnicity (vs. White), working as a frontline worker in a health or care setting (vs. not working as a frontline worker), recent travel to a place of work or study, number of public transport journeys, visits to shops and other indoor public places, travel outside of the UK, high levels of alcohol consumption (≥ 15 units per week), high body-mass index (BMI ≥ 25 kg/m^2^), sex hormone therapy (i.e. hormone replacement therapy and hormonal contraception) and use of vitamin D supplements were independently associated with higher risk of SARS-CoV-2 infection as indicated by antibody seropositivity (Table 2). By contrast, postgraduate education (vs. primary or secondary), passive smoking, high levels of light physical exercise (walking ≥10 h per week) and prescribed paracetamol use were independently associated with lower risk of SARS-CoV-2 infection. In the fully adjusted model, the associations originally observed in minimally adjusted models for generational composition of households, living with a working-age adult, and lower impact physical activity no longer achieved conventional significance (Table 2). Excluding the 796 participants with symptom-defined probable COVID-19, who did not have a positive PCR or lateral flow test result before enrolment, had little effect on our findings and associations with only 5 items were substantially attenuated in the minimally adjusted model, including environmental tobacco smoke exposure, public transport journeys, people per bedroom, dairy products intake and use of sex hormone therapy (Additional file 1: Table S4).

**Table 2:** Minimally adjusted and fully adjusted odds of seropositivity

	Seropositive participants	Minimally adjusted model^a^		Fully adjusted model^b^	
	*n*/*N* (%)	OR (95% CI)	*p* value	OR (95% CI)	*p* value
**Sociodemographic, occupational and lifestyle factors**					
Age (years)					
< 30	45/323 (13.9%)	1.00		1.00	
30 to < 40	116/651 (17.8%)	1.34 (0.92–1.95)	0.13	1.42 (0.94–2.15)	0.09
40 to < 50	241/1246 (19.3%)	1.48 (1.05–2.09)	0.03	1.50 (1.01–2.24)	0.04
50 to < 60	419/2545 (16.5%)	1.22 (0.88–1.71)	0.23	1.31 (0.89–1.93)	0.17
60 to < 70	557/4098 (13.6%)	0.98 (0.71–1.37)	0.92	1.22 (0.82–1.82)	0.34
≥ 70	318/2267 (14.0%)	1.03 (0.73–1.44)	0.89	1.41 (0.92–2.15)	0.11
*p for trend*			0.89		0.91
Sex					
Female	1177/7806 (15.1%)	1.00		1.00	
Male	519/3324 (15.6%)	1.10 (0.98–1.24)	0.10	1.06 (0.93–1.20)	0.39
Ethnicity					
White	1598/10,651 (15.0%)	1.00		1.00	
Black, African, Caribbean, or Black British	10/49 (20.4%)	1.30 (0.96–1.76)	0.09	1.24 (0.90–1.71)	0.20
South Asian^c^	34/145 (23.4%)	1.65 (1.12–2.45)	0.01	1.65 (1.10–2.49)	0.02
Mixed, multiple, or other ethnic groups	54/285 (18.9%)	1.36 (0.67–2.73)	0.40	1.14 (0.52–2.50)	0.74
Housing					
Owns own home	991/7059 (14.0%)	1.00		1.00	
Mortgage	481/2716 (17.7%)	1.14 (0.99–1.32)	0.07	1.09 (0.93–1.27)	0.28
Privately renting	108/654 (16.5%)	1.12 (0.88–1.42)	0.36	1.23 (0.95–1.58)	0.11
Renting from council	56/318 (17.6%)	1.20 (0.89–1.62)	0.24	1.32 (0.94–1.87)	0.11
Other	60/382 (15.7%)	1.09 (0.79–1.50)	0.60	1.11 (0.80–1.55)	0.53
Claiming Universal Credit					
No	1654/10,823 (15.3%)	1.00		1.00	
Yes	35/275 (12.7%)	0.73 (0.51–1.05)	0.09	0.72 (0.49–1.05)	0.09
Number of people per bedroom					
≤ 0.5	590/4382 (13.5%)	1.00		1.00	
> 0.5 to < 1	499/3085 (16.2%)	1.18 (1.04–1.35)	0.01	1.04 (0.90–1.21)	0.57
1 to < 2	559/3355 (16.7%)	1.13 (0.98–1.30)	0.09	0.97 (0.83–1.14)	0.73
≥ 2	35/237 (14.8%)	0.96 (0.66–1.41)	0.85	0.73 (0.48–1.11)	0.15
*p for trend*			0.13		0.42
Multigenerational household					
Living alone	265/1997 (13.3%)	1.00		1.00	
Single generation	925/6055 (15.3%)	1.17 (1.01–1.36)	0.03	1.14 (0.95–1.38)	0.17
Two-generation	488/2990 (16.3%)	1.19 (1.01–1.40)	0.04	1.17 (0.94–1.45)	0.15
Three-generation	18/88 (20.5%)	1.61 (0.94–2.76)	0.08	1.64 (0.91–2.96)	0.10
*p for trend*	··	··	0.03	··	0.08
Shares home with working-age adult (16–64 years)					
No	724/5353 (13.5%)	1.00		1.00	
Yes	968/5748 (16.8%)	1.21 (1.06–1.37)	0.003	1.08 (0.93–1.26)	0.32
Highest educational level attained					
Primary or secondary	198 (16.6%)	1.00		1.00	
Higher or further education (A levels or TEC)	244 (15.3%)	0.92 (0.75–1.13)	0.42	0.94 (0.76–1.17)	0.60
College or university	777 (15.7%)	0.95 (0.80–1.13)	0.54	0.94 (0.78–1.12)	0.47
Postgraduate degree	476 (14.1%)	0.83 (0.69–0.99)	0.04	0.82 (0.67–0.99)	0.04
*p for trend*			0.04		0.03
Frontline worker					
No	1369/9422 (14.5%)	1.00		1.00	
Non-health	197/1201 (16.4%)	1.13 (0.95–1.33)	0.17	1.02 (0.85–1.22)	0.86
Health or care	130/499 (26.1%)	2.02 (1.63–2.50)	< 0.001	1.86 (1.48–2.33)	< 0.001
Travel to place of work or study in past week					
No	669/5031 (13.3%)	1.00		1.00	
Yes	1014/5981 (17.0%)	1.31 (1.17–1.46)	< 0.001	1.20 (1.07–1.35)	< 0.001
Number of public transport journeys per week					
0	1502/9923 (15.1)	1.00		1.00	
1–5	135/849 (15.9)	1.24 (1.02–1.51)	0.03	1.19 (0.97–1.47)	0.09
≥ 6	55/322 (17.1)	1.32 (0.98–1.78)	0.07	1.24 (0.90–1.69)	0.18
*p for trend*			0.01		0.05
Number of visits to shops and other indoor public places per week					
0	220/1514 (14.5)	1.00		1.00	
1–2	560/3611(15.5)	1.15 (0.97–1.37)	0.10	1.12 (0.94–1.34)	0.20
2–4	413/2578 (16.0)	1.33 (1.11–1.60)	0.002	1.27 (1.05–1.53)	0.02
≥ 5	503/3413 (14.7)	1.36 (1.13–1.64)	0.001	1.29 (1.06–1.57)	0.01
*p for trend*			< 0.001		0.01
Travel outside of the UK between November 2019, and February 2021^d^					
No	926/6529 (14.2%)	1.00		1.00	
Yes	572/3507 (16.3%)	1.19 (1.07–1.34)	0.002	1.20 (1.07–1.36)	0.002
Tobacco smoking status					
Never-smoker	930/6281 (14.8%)	1.00		1.00	
Ex–smoker	690/4325 (16.0%)	1.10 (0.98–1.22)	0.09	1.05 (0.94–1.18)	0.42
Current smoker	76/524 (14.5%)	0.90 (0.70–1.16)	0.43	0.81 (0.61–1.07)	0.14
Regular environmental tobacco smoke exposure					
No	1672/10,925 (15.3%)	1.00		1.00	
Yes	23/203 (11.3%)	0.65 (0.42–1.01)	0.05	0.59 (0.37–0.95)	0.03
Alcohol (units per week)					
None	438/2986 (14.7%)	1.00		1.00	
1–7	610/3932 (15.5%)	1.11 (0.97–1.27)	0.13	1.09 (0.95–1.26)	0.22
8–14	318/2249 (14.1%)	1.00 (0.86–1.17)	0.99	1.01 (0.85–1.19)	0.92
≥ 15	330/1962 (16.8%)	1.22 (1.04–1.43)	0.02	1.23 (1.04–1.46)	0.02
*p for trend*			0.07		0.06
Light physical exercise (hours per week)					
0–4	614/3631 (16.9%)	1.00		1.00	
5–9	613/3714 (16.5%)	1.01 (0.90–1.15)	0.83	1.03 (0.90–1.17)	0.69
≥ 10	468/3764 (12.4%)	0.78 (0.68–0.89)	< 0.001	0.80 (0.70–0.93)	0.003
*p for trend*	··	··	< 0.001	··	0.003
Lower impact physical activity (hours per week)					
0	991/6272 (15.8%)	1.00		1.00	
1	322/2131 (15.1%)	0.95 (0.83–1.09)	0.46	0.97 (0.84–1.12)	0.71
≥ 2	378/2695 (14.0%)	0.87 (0.77–1.00)	0.04	0.93 (0.81–1.07)	0.33
*p for trend*	··	··	0.04	··	0.33
**Diet and supplemental micronutrient intake**					
Portions of dairy products or calcium-fortified alternatives per day					
0–1	474/2928 (16.2%)	1.00		1.00	
2	460/3254 (14.1%)	0.86 (0.75–0.99)	0.04	0.88 (0.76–1.02)	0.08
3–5	389/2650 (14.7%)	0.90 (0.78–1.04)	0.17	0.92 (0.79–1.07)	0.29
≥ 6	368/2269 (16.2%)	1.03 (0.88–1.20)	0.71	1.04 (0.89–1.22)	0.63
*p for trend*			0.71		0.62
**Medical conditions and prescribed medication use**					
BMI, kg/m^2^					
< 25	741/5424 (13.7%)	1.00		1.00	
25–30	604/3586 (16.8%)	1.27 (1.13–1.43)	< 0.001	1.25 (1.10–1.41)	< 0.001
> 30	347/2096 (16.6%)	1.19 (1.03–1.37)	0.01	1.23 (1.06–1.43)	0.01
*p for trend*			0.002		0.001
COPD					
No	1656/10,917 (15.2%)	1.00		1.00	
Yes	40/213 (18.8%)	1.36 (0.95–1.93)	0.09	1.41 (0.98–2.04)	0.07
Paracetamol					
No	1643/10,691 (15.4%)	1.00		1.00	
Yes	53/439 (12.1%)	0.75 (0.56–1.01)	0.06	0.71 (0.53–0.97)	0.03
Sex hormone therapy					
No	1551 (15.1%)	1.00		1.00	
Yes	145 (17.4%)	1.18 (0.97–1.43)	0.10	1.25 (1.02–1.52)	0.03
Vitamin D (over the counter or prescribed)					
No	1066/7300 (14.6%)	1.00		1.00	
Yes	630/3830 (16.4%)	1.10 (0.99–1.23)	0.08	1.16 (1.03–1.30)	0.01
Follow-up duration (days)		1.003 (1.002–1.004)	< 0.001	1.003 (1.003–1.004)	< 0.001

Descriptive data are *n*/*N* (%) indicating number of seropositive participants (*n*) and total per category (*N*)

*BMI* body mass index, *BTEC* Business and Technology Education Council, *COPD* chronic obstructive pulmonary disease, *OR* odds ratio

^a^Adjusted for age, sex and duration of follow-up

^b^Adjusted for all factors shown and duration of follow-up. The fully adjusted analysis includes 10,734 participants with data available for all factors

^c^South Asian indicates people who self-identified their ethnic origin as Indian, Pakistani or Bangladeshi

^d^The 1094 participants with unknown or missing travel status were included in the analysis as a separate category

**Fig. 2:**
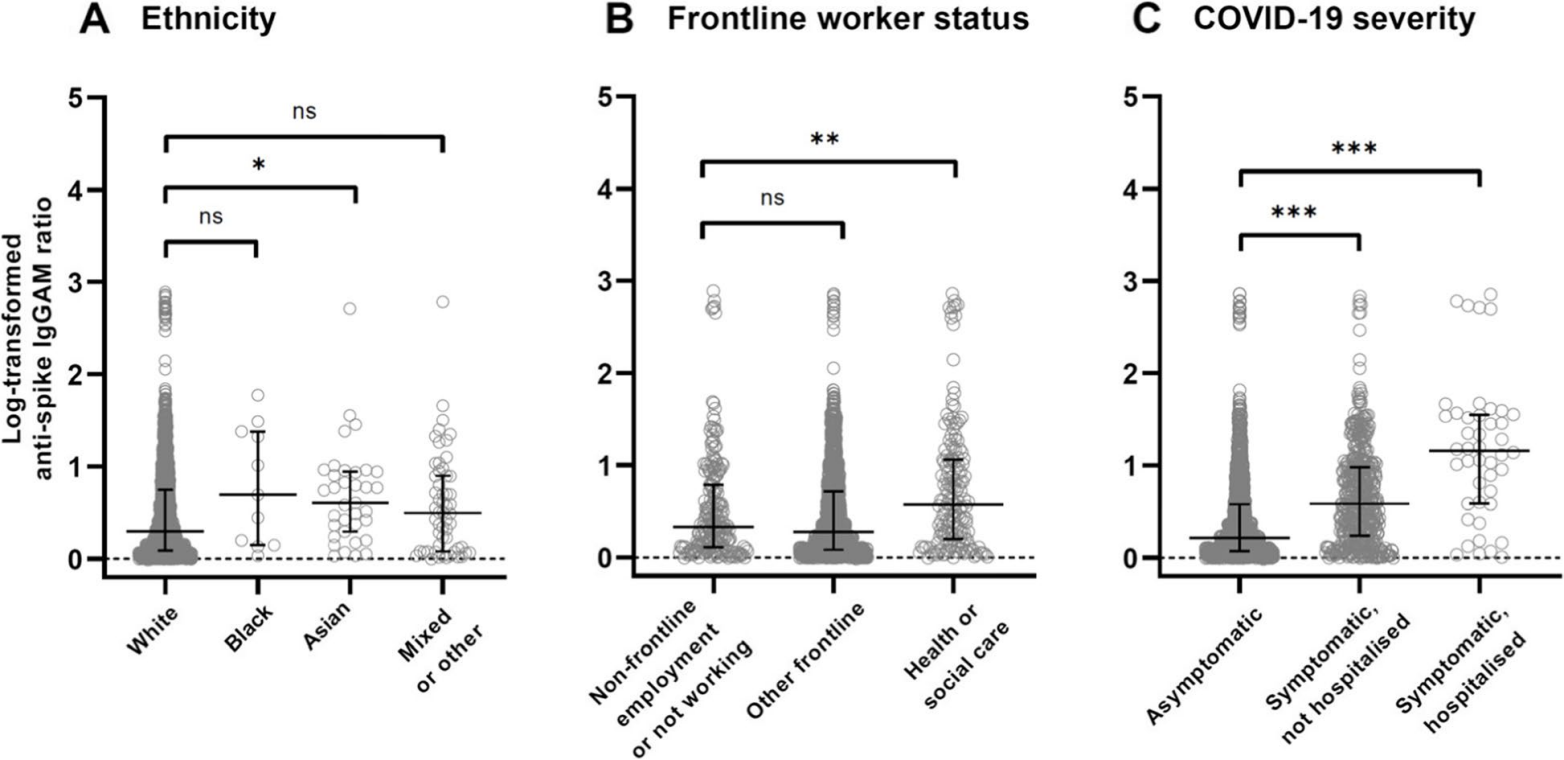
Combined IgG, IgA and IgM anti-Spike titres in seropositive participants by ethnicity, frontline worker status and COVID-19 severity. Notes: Log-transformed anti-Spike IgGAM ratios are shown for all seropositive participants (*n* = 1774) by ethnic group (**A**), frontline worker status (**B**), and COVID-19 severity (**C**), with horizontal lines showing median and IQR. **A** ‘Black’ indicates people who self-identified their ethnic origin as Black, Black British, African or Caribbean. ‘Asian’ indicates people who self-identified their ethnic origin as Indian, Pakistani, or Bangladeshi (South Asian). ‘Mixed or other’ indicates people who self-identified their ethnic origin as mixed, multiple or other. **C** COVID-19 severity was classified as ‘asymptomatic’ (non-hospitalised participants who either did not report any symptoms of acute respiratory infection, or whose symptoms were classified as having < 50% probability of being due to COVID-19), ‘symptomatic, not hospitalised’ (non-hospitalised participants reporting symptoms of acute respiratory infection that were classified as having ≥50% probability of being due to COVID-19) and ‘hospitalised’ (participants hospitalised for treatment of COVID-19). Abbreviations: *IgGAM*  IgG, IgA, and IgM combined, *p < 0.05, **p < 0.01,  ***p < 0.001, *ns*  *p* ≥ 0.05

When investigating associations with antibody titres, analysed as a continuous outcome in the subset of seropositive participants only, we found that 35 factors were independently associated with antibody titres with *p* < 0.10 after adjustment for age, sex and duration of follow-up (Table 3). The distribution of titres for three of these factors—ethnicity, frontline worker status, and COVID-19 severity—are shown in Fig. 2, with higher medians for non-White ethnicities, health or social care frontline workers, and participants who were hospitalised for treatment of COVID-19. Additional file 1: Table S5 shows factors with no evidence of association with antibody titre. When the 33 factors were included together in a fully adjusted model, we found that South Asian ethnicity (vs. White), having a mortgage (vs. owning own home), working as a frontline worker in a health or care setting, being an ex-smoker (vs. a never-smoker), visits to shops and other indoor public places, travel outside of the UK, taking multivitamin supplements, consuming at least two portions of dairy products or calcium-fortified alternatives (vs. 0–1 portions), and high BMI were associated with higher antibody titres, whereas high levels of fruit, vegetable, or salad consumption and reporting feeling anxious or depressed at baseline were associated with lower antibody titres (Table 3). P-for-trend analyses suggested higher antibody titres with increasing intake of dairy or calcium-fortified alternatives and increasing BMI and lower antibody titres with increasing fruit, vegetable or salad consumption (Table 3). The associations in minimally adjusted models with chronic obstructive pulmonary disease, poor self-reported general health and use of metformin or statins were attenuated in the fully adjusted model and were no longer statistically significant (Table 3).

**Table 3:** Minimally adjusted and fully adjusted geometric mean ratios of antibody titres in seropositive participants, with exploratory analysis of disease severity

	***n*** (%)	Minimally adjusted model^a^		Fully adjusted model^b^		Fully adjusted model plus adjustment for disease severity^**c**^	
		GMR (95% CI)	*p* value	GMR (95% CI)	*p* value	GMR (95% CI)	*p* value
**Sociodemographic, occupational and lifestyle factors**							
Age (years)							
< 30	50 (2.8%)	1.00		1.00		1.00	
30 to < 40	124 (7.0%)	0.96 (0.80–1.15)	0.68	0.95 (0.78–1.16)	0.62	0.97 (0.81–1.16)	0.71
40 to < 50	257 (14.5%)	0.98 (0.83–1.16)	0.83	1.00 (0.82–1.22)	0.97	1.05 (0.88–1.25)	0.60
50 to < 60	445 (25.1%)	0.96 (0.82–1.12)	0.60	1.00 (0.82–1.22)	1.00	1.05 (0.88–1.25)	0.59
60 to < 70	575 (32.4%)	0.94 (0.80–1.10)	0.45	1.04 (0.84–1.27)	0.74	1.11 (0.92–1.33)	0.28
≥ 70	323 (18.2%)	0.94 (0.80–1.11)	0.46	1.05 (0.85–1.31)	0.64	1.14 (0.94–1.39)	0.18
*p for trend*			0.46		0.21		0.02
Sex							
Female	1234 (69.6%)	1.00		1.00		1.00	
Male	540 (30.4%)	1.00 (0.95–1.05)	0.93	0.95 (0.90–1.00)	0.07	0.94 (0.89–1.00)	0.03
Ethnicity							
White	1672 (94.3%)	1.00		1.00		1.00	
Black, African, Caribbean or Black British	11 (0.6%)	1.11 (0.96–1.28)	0.14	1.09 (0.93–1.27)	0.30	1.07 (0.92–1.25)	0.36
South Asian^d^	35 (2.0%)	1.19 (1.01–1.40)	0.03	1.22 (1.04–1.44)	0.02	1.23 (1.04–1.47)	0.02
Mixed, multiple or other ethnic groups	56 (3.2%)	1.33 (0.93–1.91)	0.12	1.15 (0.78–1.71)	0.48	1.20 (0.81–1.77)	0.37
Housing							
Owns own home	1021 (57.6%)	1.00		1.00		1.00	
Mortgage	515 (29.0%)	1.12 (1.04–1.20)	0.002	1.08 (1.00–1.15)	0.05	1.06 (0.99–1.14)	0.08
Privately renting	112 (6.3%)	1.07 (0.96–1.20)	0.22	1.00 (0.89–1.13)	0.96	0.99 (0.89–1.10)	0.84
Renting from council	61 (3.4%)	1.05 (0.92–1.21)	0.47	1.00 (0.86–1.16)	1.00	1.01 (0.87–1.17)	0.90
Other	65 (3.7%)	1.17 (0.97–1.41)	0.09	1.14 (0.94–1.38)	0.19	1.13 (0.94–1.35)	0.20
Number of people per bedroom							
≤ 0.5	611 (34.7%)	1.00		1.00		1.00	
> 0.5 to < 1	521 (29.6%)	1.03 (0.97–1.09)	0.36	1.01 (0.95–1.07)	0.86	1.00 (0.94–1.05)	0.92
1 to < 2	593 (33.7%)	1.05 (0.99–1.12)	0.12	1.03 (0.96–1.10)	0.37	1.03 (0.96–1.09)	0.43
≥ 2	36 (2.0%)	1.26 (1.00–1.60)	0.05	1.20 (0.94–1.54)	0.15	1.22 (0.96–1.54)	0.10
*p for trend*			0.04		0.19		0.19
IMD rank							
Quartile 1 (least wealthy)	432 (24.4%)	0.99 (0.93–1.06)	0.87	0.95 (0.88–1.02)	0.16	0.96 (0.90–1.03)	0.30
Quartile 2	428 (24.2%)	1.04 (0.97–1.12)	0.26	1.01 (0.94–1.08)	0.88	1.02 (0.95–1.09)	0.65
Quartile 3	444 (25.1%)	0.93 (0.87–0.99)	0.03	0.91 (0.86–0.97)	0.004	0.93 (0.87–0.99)	0.02
Quartile 4 (most wealthy)	465 (26.3%)	1.00		1.00		1.00	
*p for trend*			0.41		0.62		0.84
Frontline worker							
No	1402 (79.0%)	1.00		1.00		1.00	
Health or social care	166 (9.4%)	1.33 (1.20–1.48)	< 0.001	1.24 (1.11–1.39)	< 0.001	1.19 (1.07–1.33)	0.001
Other	206 (11.6%)	1.08 (0.99–1.17)	0.07	1.05 (0.97–1.14)	0.26	1.03 (0.95–1.11)	0.51
Highest educational level attained							
Primary or secondary	209 (11.8%)	1.00		1.00		1.00	
Higher or further education (A levels or BTEC)	255 (14.4%)	0.93 (0.84–1.03)	0.16	0.95 (0.86–1.05)	0.29	0.97 (0.89–1.06)	0.53
College or university	805 (45.4%)	0.93 (0.85–1.01)	0.09	0.95 (0.88–1.04)	0.29	0.97 (0.90–1.06)	0.51
Postgraduate degree	504 (28.4%)	0.94 (0.85–1.02)	0.15	0.96 (0.87–1.05)	0.36	0.97 (0.89–1.06)	0.46
*p for trend*			0.22		0.50		0.53
Tobacco smoking status							
Never-smoker	969 (54.6%)	1.00		1.00		1.00	
Ex-smoker	728 (41.0%)	1.06 (1.01–1.12)	0.02	1.06 (1.00–1.11)	0.03	1.04 (0.99–1.09)	0.12
Current smoker	77 (4.3%)	0.97 (0.86–1.09)	0.59	0.90 (0.80–1.02)	0.09	0.91 (0.82–1.02)	0.10
Actual sleep (hours per night)							
≤ 5	169 (9.5%)	1.14 (1.03–1.26)	0.01	1.09 (0.98–1.22)	0.10	1.05 (0.95–1.16)	0.36
6	419 (23.6%)	1.01 (0.96–1.07)	0.80	1.00 (0.94–1.06)	0.99	1.00 (0.94–1.06)	0.99
7	728 (41.1%)	1.00		1.00		1.00	
≥ 8	457 (25.8%)	1.03 (0.97–1.09)	0.42	1.02 (0.96–1.08)	0.60	1.01 (0.95–1.07)	0.74
*p for trend*			0.03		0.30		0.70
Vigorous physical exercise (hours per week)							
0	714 (40.3%)	1.00		1.00		1.00	
1–3	635 (35.9%)	0.94 (0.89–0.99)	0.03	0.98 (0.92–1.04)	0.43	0.98 (0.93–1.04)	0.49
≥ 4	422 (23.8%)	0.97 (0.91–1.04)	0.38	1.01 (0.94–1.08)	0.87	1.01 (0.95–1.08)	0.72
*p for trend*			0.26		0.95		0.79
Light physical exercise (hours per week)							
0–4	659 (37.2%)	1.00		1.00		1.00	
5–9	631 (35.6%)	0.93 (0.88–0.98)	0.01	0.96 (0.91–1.02)	0.24	0.98 (0.93–1.04)	0.58
≥ 10	483 (27.2%)	1.00 (0.93–1.06)	0.90	1.03 (0.96–1.10)	0.44	1.03 (0.97–1.10)	0.34
*p for trend*			0.70		0.51		0.38
Lower-impact physical activity (hours per week)							
0	1048 (59.2)	1.00		1.00		1.00	
1	331 (18.7)	0.95 (0.89–1.01)	0.11	0.97 (0.91–1.03)	0.29	0.97 (0.92–1.04)	0.42
≥ 2	390 (22.0)	0.95 (0.89–1.00)	0.07	0.98 (0.92–1.05)	0.53	0.99 (0.93–1.05)	0.69
*p for trend*			0.04		0.44		0.60
Number of visits to shops and other indoor public places (per week)							
0	237 (13.4)	1.00		1.00		1.00	
1–2	591 (33.3)	1.02 (0.94–1.11)	0.57	1.06 (0.98–1.16)	0.16	1.05 (0.97–1.14)	0.22
2–4	428 (24.1)	1.08 (0.99–1.18)	0.07	1.12 (1.02–1.22)	0.02	1.12 (1.03–1.22)	0.01
≥ 5	518 (29.2)	1.12 (1.02–1.22)	0.02	1.12 (1.02–1.23)	0.02	1.12 (1.02–1.22)	0.01
*p for trend*			0.004		0.01		0.01
Number of public transport journeys per week							
0	1569 (88.7)	1.00		1.00		1.00	
1–5	139 (7.9)	1.05 (0.97–1.15)	0.24	1.04 (0.95–1.13)	0.44	1.02 (0.94–1.11)	0.60
≥ 6	60 (3.4)	1.15 (1.00–1.31)	0.05	1.09 (0.95–1.26)	0.21	1.05 (0.93–1.18)	0.44
*p for trend*			0.03		0.17		0.38
Travel to place of work or study in past week							
No	697 (39.6%)	1.00		1.00		1.00	
Yes	1064 (60.4%)	1.07 (1.01–1.12)	0.01	1.02 (0.97–1.08)	0.44	1.03 (0.98–1.08)	0.24
Travel outside of the UK between November 2019, and February 2021^e^							
No	955 (53.8%)	1.00		1.00		1.00	
Yes	602 (33.9%)	1.08 (1.02–1.13)	0.004	1.11 (1.05–1.16)	< 0.001	1.09 (1.04–1.15)	0.001
**Diet and supplemental micronutrient intake**							
Multivitamin supplement							
No	1377 (77.6%)	1.00		1.00		1.00	
Yes	397 (22.4%)	1.08 (1.02–1.16)	0.01	1.08 (1.01–1.15)	0.02	1.09 (1.02–1.16)	0.009
Iron (only) supplement							
No	1713 (96.6%)	1.00		1.00		1.00	
Yes	61 (3.4%)	1.17 (0.99–1.38)	0.07	1.16 (0.99–1.37)	0.07	1.15 (0.98–1.35)	0.09
Zinc (only) supplement							
No	1673 (94.3%)	1.00		1.00		1.00	
Yes	101 (5.7%)	0.91 (0.83–1.00)	0.06	0.94 (0.85–1.03)	0.18	0.92 (0.84–1.02)	0.10
Selenium (only) supplement							
No	1754 (98.9)	1.00		1.00		1.00	
Yes	20 (1.1)	0.87 (0.73–1.03)	0.10	0.86 (0.72–1.03)	0.11	0.86 (0.74–1.01)	0.07
Fruit, vegetable, or salad intake per day							
0–2	266 (15.1%)	1.00		1.00		1.00	
3–4	559 (31.7%)	0.94 (0.87–1.02)	0.12	0.94 (0.86–1.02)	0.12	0.94 (0.87–1.01)	0.11
5	374 (21.2%)	0.93 (0.86–1.02)	0.11	0.92 (0.85–1.01)	0.07	0.92 (0.85–1.00)	0.06
≥ 6	567 (32.1%)	0.92 (0.86–1.00)	0.05	0.91 (0.84–0.99)	0.03	0.90 (0.84–0.98)	0.01
*p for trend*			0.09		0.05		0.02
Portions of dairy products or calcium-fortified alternatives per day							
0–1	491 (27.8%)	1.00		1.00		1.00	
2	485 (27.4%)	1.07 (1.00–1.14)	0.04	1.08 (1.01–1.15)	0.02	1.09 (1.02–1.16)	0.01
3–5	407 (23.0%)	1.10 (1.03–1.19)	0.01	1.12 (1.04–1.20)	0.002	1.10 (1.03–1.18)	0.01
≥ 6	386 (21.8%)	1.06 (0.99–1.14)	0.08	1.09 (1.01–1.17)	0.03	1.08 (1.00–1.16)	0.04
*p for trend*			0.04		0.01		0.02
**Medical conditions and medication use**							
Self-reported general health							
Excellent	341 (19.2%)	1.00		1.00		1.00	
Very good	701 (39.5%)	1.03 (0.96–1.10)	0.42	0.99 (0.93–1.06)	0.83	0.99 (0.93–1.06)	0.80
Good	474 (26.7%)	1.02 (0.95–1.09)	0.67	0.98 (0.91–1.06)	0.61	0.97 (0.90–1.05)	0.46
Fair	198 (11.2%)	1.07 (0.97–1.18)	0.18	1.01 (0.91–1.12)	0.80	0.99 (0.90–1.09)	0.86
Poor	59 (3.3%)	1.18 (1.00–1.39)	0.05	1.14 (0.95–1.36)	0.17	1.05 (0.87–1.27)	0.62
*p for trend*			0.07		0.47		0.96
BMI, kg/m^2^							
< 25	764 (43.2%)	1.00		1.00		1.00	
25–30	626 (35.4%)	1.05 (1.00–1.11)	0.07	1.04 (0.98–1.10)	0.19	1.03 (0.98–1.09)	0.28
> 30	379 (21.4%)	1.11 (1.04–1.19)	0.003	1.10 (1.02–1.19)	0.01	1.07 (1.00–1.15)	0.06
*p for trend*			0.002		0.008		0.05
Diabetes or pre-diabetes							
No diabetes	1636 (92.3%)	1.00		1.00		1.00	
Pre-diabetes	52 (2.9%)	1.03 (0.89–1.19)	0.70	0.97 (0.85–1.11)	0.65	0.99 (0.87–1.13)	0.88
Type 1	12 (0.7%)	1.00 (0.77–1.30)	0.99	0.95 (0.71–1.27)	0.73	0.99 (0.73–1.34)	0.96
Type 2	72 (4.1%)	1.14 (0.98–1.32)	0.09	0.97 (0.82–1.15)	0.71	0.96 (0.82–1.12)	0.59
Asthma/atopy							
No/no	1178 (66.4)	1.00		1.00		1.00	
No/yes	305 (17.2)	0.95 (0.89–1.01)	0.10	0.95 (0.89–1.01)	0.09	0.95 (0.90–1.00)	0.07
Yes/no	145 (8.2)	1.00 (0.91–1.10)	0.98	0.97 (0.88–1.07)	0.55	0.95 (0.87–1.05)	0.33
Yes/yes	146 (8.2)	0.99 (0.91–1.08)	0.82	0.99 (0.90–1.09)	0.87	1.00 (0.91–1.10)	0.97
COPD							
No	1728 (97.4%)	1.00		1.00		1.00	
Yes	46 (2.6%)	1.22 (1.01–1.49)	0.04	1.12 (0.93–1.35)	0.23	1.08 (0.91–1.29)	0.39
Reported feeling anxious or depressed at baseline							
No	1294 (73.0%)	1.00		1.00		1.00	
Yes	479 (27.0%)	0.95 (0.90–1.00)	0.07	0.92 (0.87–0.97)	0.003	0.91 (0.87–0.97)	0.002
Anticholinergics							
No	1685 (95.0%)	1.00		1.00		1.00	
Yes	89 (5.0%)	1.16 (1.01–1.34)	0.04	1.15 (0.99–1.32)	0.06	1.16 (1.00–1.33)	0.04
Beta blockers							
No	1659 (93.5%)	1.00		1.00		1.00	
Yes	115 (6.5%)	1.12 (1.01–1.25)	0.04	1.09 (0.98–1.22)	0.10	1.12 (1.01–1.24)	0.03
Metformin							
No	1720 (97.0%)	1.00		1.00		1.00	
Yes	54 (3.0%)	1.23 (1.04–1.47)	0.02	1.13 (0.91–1.42)	0.28	1.17 (0.95–1.43)	0.14
Statins							
No	1487 (83.8%)	1.00		1.00		1.00	
Yes	287 (16.2%)	1.07 (0.99–1.15)	0.07	1.04 (0.96–1.13)	0.31	1.04 (0.97–1.13)	0.28
Follow-up duration (days)		0.001 (0.001–0.001)	< 0.001	0.001 (0.001–0.002)	< 0.001	0.001 (0.001–0.001)	< 0.001
COVID-19 severity^g^							
Asymptomatic	1242 (70.6%)	1.00				1.00	
Symptomatic, not hospitalised	473 (26.9%)	1.29 (1.22–1.36)	< 0.001			1.25 (1.18–1.33)	< 0.001
Hospitalised	44 (2.5%)	2.13 (1.70–2.67)	< 0.001			1.92 (1.54–2.40)	< 0.001
*p for trend*			< 0.001				< 0.001

Minimally adjusted models included 1774 participants, except for a few items that included sample sizes were slightly lower (ranged from 1759 to 1773) because of missing values. The fully adjusted analyses included 1707 participants with data available for all factors, and 1694 participants after adjustment for COVID-19 severity

*BMI* body mass index, *BTEC* Business and Technology Education Council, *COPD* chronic obstructive pulmonary disease, *GMR* geometric mean ratio, *IMD* index of multiple deprivation

^a^Adjusted for age, sex and duration of follow-up

^b^Adjusted for duration of follow-up and all factors shown except symptom severity

^c^Adjusted for duration of follow-up and all factors shown

^d^South Asian indicates people who self-identified their ethnic origin as Indian, Pakistani or Bangladeshi

^e^The 217 participants with unknown or missing travel status were included in the analysis as a separate category to ensure greater power

^g^COVID-19 severity was classified as ‘asymptomatic’ (non-hospitalised participants who either did not report any symptoms of acute respiratory infection, or whose symptoms were classified as having <50% probability of being due to COVID-19), ‘symptomatic, not hospitalised’ (non-hospitalised participants reporting symptoms of acute respiratory infection that were classified as having ≥50% probability of being due to COVID-19) and ‘hospitalised’ (participants hospitalised for treatment of COVID-19)

The addition of COVID-19 severity to our model of determinants of antibody titres in seropositive participants attenuated the associations observed for BMI and smoking status, but significant associations remained for all other variables (Table 3). Inclusion of the severity variable also led to a weak inverse association between male sex and antibody titres (GMR 0.94 [95% CI 0.89–1.00]; *p* = 0.03). Also, use of beta blockers (1.12 [1.01–1.24]; *p* = 0.03) and anticholinergics (1.16 [1.00–1.33]; *p* = 0.04) were positively associated with antibody titres in this model (Table 3). *P*-for-trend analyses suggested lower antibody titres with increasing fruit, vegetable and salad intake and higher titres with increasing intake of dairy or calcium-fortified alternatives and increasing age (Table 3). The variance in antibody titre explained by the fully adjusted model was increased from 14.5% (*R*^2^ = 0.1447) to 20.5% (*R*^2^ = 0.2047) after including disease severity as a covariate.

## Discussion

In this large, prospective, population-based serological study, we explored determinants of SARS-CoV-2 seropositivity and antibody titres, evaluating more than 80 potential sociodemographic, clinical and behavioural risk factors. We found that five factors—South Asian ethnicity, frontline occupation in health or social care, number of visits to shops and other indoor public places, international travel, and high BMI—were strongly associated both with higher risk of SARS-CoV-2 seropositivity among all participants and with higher antibody titres in the subset of seropositive participants. Lower levels of educational attainment and light physical exercise, and higher levels of public transport use and alcohol consumption, were found to associate with higher risk of SARS-CoV-2 seropositivity. Lower intake of dairy or calcium-fortified alternatives and higher intake of fruit, vegetables and salad were associated with lower antibody titres, as was reporting anxiety or depression. Importantly, most factors associated with antibody titres in seropositive participants retained significance after adjusting for disease severity.

Our results support previous studies that have found increased risk of SARS-CoV-2 seropositivity for healthcare workers [3, 4, 12], people of Asian ethnicity [3, 5, 12] and people with lower educational attainment [3, 4]. Non-White race/ethnicity has previously been highlighted as a determinant of both SARS-CoV-2 seropositivity [22–24] and antibody titres [25], but questions remained over residual confounding [22]. Despite including a wide range of potential confounders, point estimates for all non-White participants remained elevated in both our seropositivity and titre analyses, and significantly so for South Asian participants, emphasising the need to further investigate the underlying biological or social factors driving this disparity. While we did not confirm increased seropositivity for Black participants, we lacked statistical power, as they represented only 0.4% of the cohort.

We identified two novel modifiable lifestyle factors associated with SARS-CoV-2 seropositivity: alcohol consumption and light physical exercise. High levels of alcohol intake are known to negatively affect immune response through several mechanisms [26], which supports our finding of higher risk among participants consuming more than 15 units of alcohol a week. By contrast, we observed lower risk among participants doing more than 10 h of light physical exercise per week. It has been speculated that there is a J-shaped relationship between exercise load and susceptibility to infection, whereby moderate exercise can improve immune response, but prolonged, high-intensity exercise can increase susceptibility to infection [27]. This curve might explain why we did not see similar benefits for vigorous physical activity.

Our finding that use of vitamin D supplements was associated with higher risk of seropositivity contrasts with a previous study, which found lower risk in a univariable model and no association after adjusting for confounders [23]; randomised controlled trials are needed to resolve questions around potential effects of vitamin D supplements on susceptibility to SARS-CoV-2. We found no associations for frontline workers not based in health or social care, at odds with previous findings [11, 12]. We also did not observe associations between seropositivity and age or sex, unlike other studies [5, 23, 24, 28, 29]. This may reflect the fact that we adjusted for more potential confounders than most of these studies and included behaviours that reflect social mixing and influence exposure to infectious index cases.

The strongest association with antibody titre was for disease severity, which explained a further 6% of variance when added to our full model, including 35 factors explaining 14.5% of variance in antibody titre together. After adjustment for disease severity, we uncovered ten factors associated with higher titres (greater age, South Asian ethnicity, higher BMI, working as a frontline worker in a health or care setting, greater number of visits to shops or other indoor places, international travel, taking multivitamin supplements, increased consumption of dairy products or calcium-fortified alternatives, and use of beta blockers and anticholinergic medications) and three associated with lower titres (male sex, high levels of fruit, vegetable, or salad consumption; and reporting feeling anxious or depressed at baseline). Different mechanisms may explain these associations. High intensity and frequency of exposure could be a cause of elevated antibody titres in participants who visited indoor public places or travelled abroad more frequently and in frontline health and social care workers [30], supported by previous findings of higher titres in healthcare workers [14]. Alternatively, greater immune reactivity could be a cause of higher titres, as seen in female participants, who generally have stronger innate and adaptive immune responses than males [31]. Diet and nutrition are known to affect immune responses [32] and thus might explain the higher titres observed with use of multivitamin supplements and higher levels of dairy intake (potentially reflecting higher calcium intakes) [33]. However, little evidence is available for the effect of vitamin supplementation in suboptimal rather than micronutrient-deficient diets [32], and after adjustment, we found no associations between intake of any individual vitamin supplements and antibody titres. The negative association with fruit, vegetable, and salad consumption was observed for the highest level of intake only (≥ 6 portions a day); however, despite 40% of vegan or vegetarian participants being included in that category, neither diet type was found to be associated with antibody titres, suggesting it is not the result of a restricted diet. Our finding of a significant positive dose–response relationship between age and antibody titres after adjustment for disease severity supports findings from other studies [13, 34, 35].

This study has several strengths. Use of serology to measure SARS-CoV-2 infection reduces collider bias, as serology testing was offered to all participants enrolled in COVIDENCE UK, in contrast to results from external routine testing that had limited availability, particularly at the start of the pandemic. Serology testing has also allowed us to better quantify the risk of SARS-CoV-2 infection by capturing previous infections that were asymptomatic or unconfirmed. A further strength is the use of an assay with high sensitivity and specificity that targets three different types of antibody [36], increasing the probability of identifying a past infection. Additionally, we used dried blood spots for our sampling, which have been found to reduce processing failures compared with microtubes [37], which are currently used by large seroprevalence surveys [38]. The prospective nature of our study reduces the potential for reverse causation explaining our findings, and the granularity of our questionnaire allowed us to explore potential determinants and confounders that other studies have not investigated.

Our study also has some limitations. First, COVIDENCE UK is a self-selected cohort, and thus several groups—such as people younger than 30 years, people of lower socioeconomic status, and non-White ethnic groups—are under-represented. This particularly affected our power to investigate outcomes for Black participants, who have been found to be at higher risk of SARS-CoV-2 infection [4, 5, 12] and adverse outcomes [22] than White people. However, insufficient representativeness in a cohort does not preclude identification of causal associations, and self-selection may result in better response to follow-up [39]. Second, as we included asymptomatic infections in our titre analysis, we were not able to adjust for timing of infection onset, preventing us from capturing the effects of temporal changes in antibody responses. As 40% of our seropositive participants did not experience symptoms suggestive of COVID-19 or provide a symptom onset date, excluding them would have greatly reduced the power and generalisability of our analysis. Third, as with any observational study, we cannot exclude the possibility that some of the associations we report might be explained by residual or unmeasured confounding. For example, the finding that passive smoking but not active smoking was associated with a reduced risk of seropositivity compared with never-smokers should be treated with caution, unless a plausible protective mechanism can be found. However, we have minimised potential confounding by adjusting for a comprehensive list of putative risk factors. Finally, we explored numerous potential associations and cannot discount the possibility that some may have achieved statistical significance as a result of type 1 error. We therefore hope that future studies will test for the associations we report to determine whether they can be replicated in different populations.

## Conclusions

This prospective serological study shows that people of South Asian ethnicity, frontline workers in health or social care, people with high BMI, and those who had more visits to indoor public places or who had travelled abroad were at higher risk of SARS-CoV-2 seropositivity, after robust adjustment for confounders. Moreover, among seropositive participants, all of these factors associated independently with higher antibody titres, regardless of disease severity. We additionally show that higher alcohol consumption and lower light physical exercise, both modifiable lifestyle factors, are associated with higher risk of seropositivity. Future research should focus on modifiable risk factors for seropositivity, as well as determinants of antibody titres and other correlates of protection after SARS-CoV-2 infection, to better understand which groups are most at risk of reinfection and what preventive measures might be taken.

## Supplementary Information

**Additional file 1: Table S1.** Baseline questions. **Table S2.** Monthly follow-up questions. **Table S3.** Factors with no evidence of association with seropositivity for COVID-19 in minimally adjusted model. **Table S4.** Seropositivity sensitivity analysis excluding participants with symptom-defined probable COVID-19. **Table S5.** Factors with no evidence of association with antibody titres in seropositive participants in minimally adjusted model.

## Data Availability

De-identified participant data will be made available upon reasonable request to the corresponding author.

## Abbreviations

aGMR: Adjusted geometric mean ratio
aOR: Adjusted odds ratio
BMI: Body mass index
BTEC: Business and Technology Education Council
CI: Confidence interval
COPD: Chronic obstructive pulmonary disease
COVID-19: Coronavirus disease 2019
ELISA: Enzyme-linked immunosorbent assay
HIV: Human immunodeficiency virus
IgGAM: Combined IgG, IgA, and IgM responses
IMD: Index of multiple deprivation
IQR: Interquartile range
PBS: Phosphate-buffered saline
RT-PCR: Reverse transcription polymerase chain reaction
SARS-CoV-2: Severe acute respiratory syndrome coronavirus 2
SD: Standard deviation
TBS: The Binding Site
